# *Wolbachia* Divergence and the Evolution of Cytoplasmic Incompatibility in *Culex pipiens*

**DOI:** 10.1371/journal.pone.0087336

**Published:** 2014-01-31

**Authors:** Célestine M. Atyame, Pierrick Labbé, Emilie Dumas, Pascal Milesi, Sylvain Charlat, Philippe Fort, Mylène Weill

**Affiliations:** 1 CNRS, University Montpellier 2, ISEM - UMR 5554, Montpellier, France; 2 CNRS, University Lyon 1, LBBE - UMR 5558, Villeurbanne, France; 3 CNRS, University Montpellier 2, CRBM - UMR 5237, Montpellier, France; International Atomic Energy Agency, Austria

## Abstract

Many insect species harbor *Wolbachia* bacteria that induce cytoplasmic incompatibility (CI), i.e. embryonic lethality in crosses between infected males and uninfected females, or between males and females carrying incompatible *Wolbachia* strains. The molecular mechanism of CI remains unknown, but the available data are best interpreted under a *modification*–*rescue* model, where a *mod* function disables the reproductive success of infected males’ sperm, unless the eggs are infected and express a compatible *resc* function. Here we examine the evolution of CI in the mosquito *Culex pipiens*, harbouring a large number of closely related *Wolbachia* strains structured in five distinct phylogenetic groups. Specifically, we used a worldwide sample of mosquito lines to assess the hypothesis that genetic divergence should correlate with the divergence of CI properties on a low evolutionary scale. We observed a significant association of *Wolbachia* genetic divergence with CI patterns. Most *Wolbachia* strains from the same group were compatible whereas those from different groups were often incompatible. Consistently, we found a strong association between *Wolbachia* groups and their *mod-resc* properties. Finally, lines from the same geographical area were rarely incompatible, confirming the conjecture that the spatial distribution of *Wolbachia* compatibility types should be constrained by selection. This study indicates a clear correlation between *Wolbachia* genotypes and CI properties, paving the way toward the identification of the molecular basis of CI through comparative genomics.

## Background

*Wolbachia* bacteria are among the most common endosymbionts of arthropods and filarial nematodes [1]–[4]. Maternally inherited through the egg cytoplasm, they manipulate their host reproduction by various means, all increasing the proportion of infected females over generations, thus favoring their own dispersal [5], [6]. The most commonly described *Wolbachia*-induced phenotype in arthropods is cytoplasmic incompatibility (CI) [2]. CI is a form of conditional sterility resulting in embryonic lethality in diploid organisms [7] or in the production of male offspring in some haplo-diploid species [8]. CI occurs either in crosses between *Wolbachia* infected males and uninfected females or in crosses between males and females infected with incompatible strains of *Wolbachia*. CI is termed bidirectional if the death of embryos occurs in the two reciprocal crosses, or unidirectional, if only one cross is incompatible.

The molecular mechanisms underlying CI are currently unknown. However, cytological studies commonly show paternal chromosome condensation failure and abnormal segregation in the first mitotic division, leading to embryonic death [9]. These observations are currently best interpreted under a toxin/antitoxin model [10], [11]. According to this model, *Wolbachia* in males modify the sperm (the so-called modification, or *mod* factor) by depositing a kind of “toxin” during its maturation. *Wolbachia* in females, on the other hand, deposit an “antitoxin” (the *rescue*, or *resc* factor) in the eggs, so that the offspring of infected females can develop normally. The simple compatibility patterns seen in several insect host species [12]–[14] have initially led to the view that CI relied on a single pair of *mod*/*resc* genes. However, more complex patterns, such as those described in the mosquito *Culex pipiens* [15]–[17] suggest that CI is controlled by multiple *mod*/*resc* factors that interact in complex ways [17]–[20]. Here we are interested in the processes that shape the evolution of compatibility types within *Cx. pipiens.*

Mosquitos of the *Cx. pipiens* complex are infected by a variety of strains from the *w*Pip *Wolbachia* clade. This diversity represents an ideal model to study the relationship between *Wolbachia* genetics and CI properties for the following reasons: (1) all *w*Pip strains share a monophyletic origin within the *Wolbachia* B group as evidenced by *Wolbachia* multilocus strain typing methodology [21]; (2) the recent sequencing of fast evolving genes indicates that five phylogenetic groups can be distinguished within the *w*Pip clade, referred to as *w*Pip-I to *w*Pip-V, [21], [22]; (3) multiple infections have never been evidenced despite the use of sensitive polymorphic markers [17], [21]–[26]; (4) finally, this system is characterized by an unrivalled variability of compatibility types, including compatible as well as uni- and bi-directionally incompatible lines [15]–[17], [27], [28]. Such a variability relies on the rapid diversification of crossing types [29] and is independent from the nuclear background [16], [17], [30] or from other inherited symbionts known to manipulate insect reproduction [29].

In this study, we took opportunity of the recently worked-out *w*Pip phylogeny to address the correlation between *w*Pip genetic divergence and crossing properties.

## Methods

### Mosquito Collection and Isofemale Lines Maintenance

*Culex pipiens* larvae and pupae were collected in three countries (Tunisia in 2007, 2008 and 2009, Algeria in 2006 and 2008 and in New Mexico in 2012). None of the samples in any location were collected in protected areas, and these field studies did not involve endangered or protected species. No specific permission was required to collect mosquito larvae in public areas, and when collected on private land or in private residences, the owners or residents gave permission for the study to be conducted on their land or in their residences. Samples were reared to adulthood in laboratory and females were blood-fed to establish isofemale lines. Each egg raft (containing 100–300 eggs) was individually isolated for hatching and *Wolbachia* was genotyped by analysing two first-instar larvae (L1) (see below). For each locality, two isofemale lines carrying the same *w*Pip group were maintained, whenever possible, to constitute replicates. Using the same procedure, two isofemale lines were established from samples collected in China in 2003. A total of 29 isofemale lines were thus established for the present study. In addition, 22 isofemale lines from laboratory stocks of various geographical origins were also used. They include one line from Tunisia [24]; two lines from La Reunion island [17]; four lines from Lebanon; four lines from Mauritius [22]; four lines from Mayotte [22]; three lines from France [22], [24]; two lines from California [24], [31]; one line from Italy [32] and one line from Turkey [24] (Table S1 in File S1). Isofemale lines were reared in 65 dm^3^ screened cages kept in a single room at 22 to 25°C, under a 12-h light/12-h dark cycle. Larvae were fed with a mixture of shrimp powder and rabbit pellets, while adults were fed with a honey solution.

### *w*Pip Strain Identification

Mosquito DNA was extracted using a CetylTrimethylAmmonium Bromide protocol (CTAB) [33]. The genotyping of *w*Pip strains infecting isofemale lines was performed through PCR/RFLP tests on two ankyrin-domain genes, *ank2* and *pk1* [26], [34]. Both genes clearly differentiate the five previously identified *w*Pip groups (*w*Pip-I to *w*Pip-V) [21]. The *HinfI* restriction enzyme was used for the *ank2* gene, whereas the discrimination of the five *w*Pip groups with the *pk1* gene was performed using a combination of *TaqI* and *PstI* restriction enzymes [22]. Digested DNA fragments were separated on 2% agarose gel electrophoresis.

### Crossing Properties

Isofemale lines were reared for at least four generations before crossing to allow acclimation to laboratory conditions and to optimize mating and blood feeding. Reciprocal crosses were performed using 25–50 virgin females and an equivalent number of males. All individuals were 2–5 days old. Females were allowed to blood-feed five days after caging and their egg rafts were collected five days later and stored individually until hatching. Crossing relationships between isofemale lines were determined by examining eggs’ hatching rate (HR) under a binocular microscope. All unhatched egg rafts were checked for fertilization through observation of embryonic development as described by Duron & Weill [35].

The crossing relationships between two given isofemale lines were categorized as follows:

Compatible (C) when HR was >90% in the two reciprocal crosses;
Incompatible (IC), with two CI patterns: uni-directionally incompatible crosses (UIC), when HR was 0% in one of the reciprocal crosses and >90% in the other, and bi-directionally incompatible crosses (BIC), when HR was 0% in both reciprocal crosses. Note that crosses with intermediate HR (90%> HR >0%) represented less than 5% of all crosses and were discarded from the analysis.

We examined the variability in the crossing properties of isofemale lines through reciprocal crossing of each line with 4 reference isofemale lines: Lv (*w*Pip-II), Mc and Sl (*w*Pip-III) and Is (*w*Pip-IV), already used as references in a previous investigation [17]. For each studied line, the outcome of crossing males with females of the 4 reference lines defines the male crossing type or CT (*mod* ability) while the outcome of crossing of females with males of the 4 reference lines defines the female CT (*resc* ability). The resulting cytotypes, referred to herein as 4-ref-cytotypes (4RCTs), correspond to the combination of male and female CTs (8 crosses for each one).

### Statistical Analyses

#### IC frequency in intra-group and in inter-group crosses

Using the data shown in Table 1, we performed two analyses to understand how phylogenetic groups affect compatibility, first between C *vs* IC crosses and second by distinguishing among IC crosses between UIC and BIC.

**Table 1:** Crossing relationships of *Culex pipiens* isofemale lines according to *w*Pip groups.

Categories of crosses		Total	C		UIC		BIC	
			N	Mean (SD)	N	Mean (SD)	N	Mean (SD)
Within *w*Pip groups	*w*Pip-I/*w*Pip-I	168	160	0.94 (0.016)	8	0.06 (0.015)	0	0
	*w*Pip-II/*w*Pip-II	6	6		0		0	
	*w*Pip-III/*w*Pip-III	9	5		4		0	
	*w*Pip-IV/*w*Pip-IV	19	19		0		0	
	*w*Pip-V/*w*Pip-V	1	1		0		0	
	Total	203	191		12		0	
Between *w*Pip groups	*w*Pip-I/*w*Pip-II	45	29	0.45 (0.03)	11	0.32 (0.03)	5	0.23 (0.026)
	*w*Pip-I/*w*Pip-III	67	41		26		0	
	*w*Pip-I/*w*Pip-IV	36	0		2		34	
	*w*Pip-I/*w*Pip-V	10	10		0		0	
	*w*Pip-II/*w*Pip-III	14	8		6		0	
	*w*Pip-II/*w*Pip-IV	22	0		16		6	
	*w*Pip-II/*w*Pip-V	7	4		3		0	
	*w*Pip-III/*w*Pip-IV	42	15		17		10	
	*w*Pip-III/*w*Pip-V	6	6		0		0	
	*w*Pip-IV/*w*Pip-V	3	0		0		3	
	Total	252	113		81		58	

Total indicates the total number of reciprocal crosses performed to established CI patterns, and N the number of crosses that were compatible (C), uni-directionally incompatible (UIC) and bi-directionally incompatible (BIC). SD = standard deviation. In incompatible crosses, HR  = 0%; in compatible crosses, HR >90%. For more details about crosses within *w*Pip groups see Tables S2, S3, S4, S5 and S6 in File S1 whilst for crosses between *w*Pip groups see Tables S7, S8, S9 and S10 in File S1.

- *C vs IC crosses*

We first tested if the probability for two strains to be compatible was different if they belonged to the same *w*Pip group (Intra*w*Pip) or if they were from two different groups (Inter*w*Pip). We computed the generalized linear model (GLM) PropIC = Cross+ε, where PropIC is a two-level variable corresponding to the proportions of IC and C crosses (with IC = UIC+BIC), and Cross a two-level factor indicating whether the crosses are intra or inter *w*Pip groups. ε is the error parameter, following a binomial distribution to take over-dispersion into account, if present. We tested the significance of the Cross factor using likelihood ratio tests (LRT), as described in Crawley [36].

We then tested just for an Intra*w*Pip group effect on PropIC: we used the same model and procedures as above, with Cross being a five-level factor (corresponding to the five *w*Pip groups).

Finally, in Inter*w*Pip crosses, we tested whether PropIC of a given *w*Pip group depends on the *w*Pip group it was crossed with. We again used the same model and procedures as above, with Cross being a ten-level factor (corresponding to the ten possible *w*Pip_i_ x *w*Pip_j_ crosses between two of the five *w*Pip groups).

- *C vs UIC vs BIC crosses*

We then tested whether the probability for two strains to be bi-directionally rather than uni-directionally incompatible was different in Inter*w*Pip than in Intra*w*Pip crosses (i.e. dividing IC crosses between UIC and BIC). We computed the multinomial log-linear model PropIC = Cross+ε. PropIC is a three-level variable corresponding to the proportions of C, UIC and BIC crosses and Cross a two-level factor (Intra*w*Pip *vs* Inter*w*Pip). ε is the error parameter, following a multinomial distribution. We tested the significance of the Cross factor using LRT as above.

As above, we then tested for a *w*Pip group effect (considering only the Intra*w*Pip crosses) and for an Inter*w*Pip-cross effect on PropIC, using multinomial log-linear models instead of GLM.

#### Distribution of the 4-ref-cytotypes among the wPip groups

We performed a Fisher’s exact test [37] to test for independence between 4RCTs and *w*Pip groups. We next used pairwise comparisons using Fisher’s exact test to compare the 4RCT distributions between *w*Pip groups. P-values were corrected using Hommel’s sequential Bonferroni correction to take multiple testing into account [38].

## Results and Discussion

The purpose of this study was to examine the correlation between genetic divergence and compatibility among *w*Pip strains, motivated by our recent work showing a monophyletic origin of *w*Pip strains and their organization into five genetic groups [21]. We examined a large dataset of crosses between *Cx. pipiens* isofemale lines from different geographic origins, infected either with strains from the same *w*Pip group (Intra*w*Pip crosses) or with strains from different *w*Pip groups (Inter*w*Pip crosses). All *Wolbachia* strains were unambiguously assigned to one *w*Pip group using the PCR/RFLP assay on the two ankyrin-domain genes, *ank2* and *pk1* described in Dumas et al. [22] (see Methods).

We analyzed crosses of 72 isofemale lines infected with *w*Pip strains from various groups and collected in 18 countries: 35 *w*Pip-I from ten countries (Benin, Tunisia, Philippines, Greece, France [metropolitan, Reunion and Mayotte islands], Spain, Lebanon and Mauritius), four *w*Pip-II from three countries (Australia, France and Cyprus), six *w*Pip-III from three countries (California, New Mexico and France), 24 *w*Pip-IV from five countries (Algeria, Tunisia, Turkey, Italy and China) and three *w*Pip-V from two countries (China and Philippines) (Table S1 in File S1). A total of 455 reciprocal crosses (i.e. 910 crosses) including 260 new reciprocal crosses (i.e. 520 crosses) and 195 reciprocal crosses (i.e. 390 crosses) from previous surveys [16], [17], [26] were examined.

### Compatibility among *w*Pip Strains Correlates with their Genetic Relatedness

**Figure 1:**
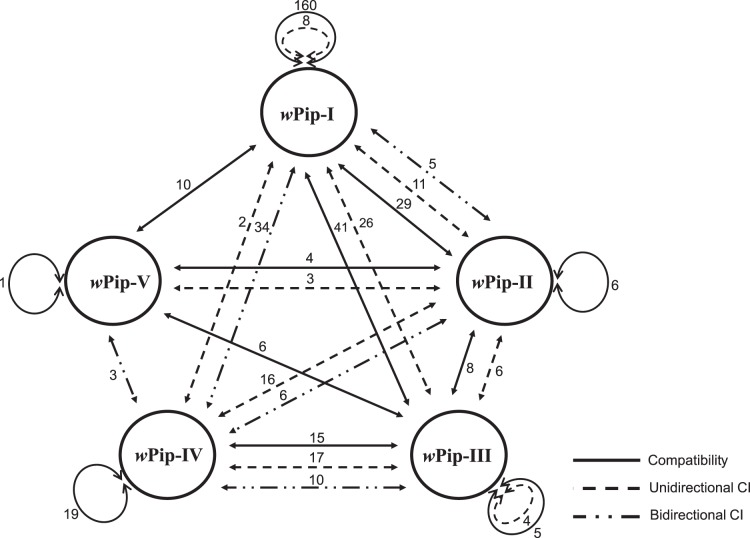
Schematic representation of the crossing relationships between *Culex pipiens* lines infected with different *w*Pip groups. Numbers indicates the number of reciprocal crosses analyzed. In all compatible crosses, hatching rate (HR) >90% and in incompatible crosses, HR  = 0%.

To the noticeable exception of the *w*Pip-III group, most crosses involving lines infected with strains from the same *w*Pip group (Intra*w*Pip crosses) were compatible (Figure 1 and Table 1). CI in the *w*Pip-I group occurred in less than 5% crosses (8 out of 168). However, intragroup CI was not found in crosses between strains of the same area and only affected strains from different geographic origins (Table 2). This supports the theoretical prediction that because of selection, only compatible *Wolbachia* strains can stably coexist in panmictic host populations [39]. A striking case of CI pattern is the Is line, infected with a *w*Pip-IV strain and long known to induce CI when crossed with most other lab lines [16], [17], [40]; however, crosses between Is and other *w*Pip-IV-infected lines were all compatible (Table 1 and Table S5 in File S1).

**Table 2:** Crossing relationships between isofemale lines infected with strains from the *w*Pip-I group and from different geographic origins.

*w*Pip-I females		*w*Pip-I males																			
		La Réunion		Mauritius				Mayotte				Tunisia						Lebanon			
		Pie-11	Leu-118	Mau-2	Mau-4	Mau-5	Mau-7	May-4	May-5	May-6	May-17	Tn	Sok	Zer11-1	Zer11-2	AinT11-1	AinT11-2	Lib-1	Lib-2	Lib-3	Lib-4
La Réunion	Pie-11		C (8)*	C (16)	C (21)	C (17)	C (18)	C (24)			C (24)	C (28)	C (20)	C (21)	C (21)	C (24)	C (20)	C (18)	C (24)	IC (20)	C (24)
	Leu-118	C (8)*		C (22)	C (24)	C (24)	C (21)	C (23)			C (18)	C (24)	C (18)	C (25)	C (24)	C (24)	C (15)	C (24)	C (21)	C (18)	C (18)
Mauritius	Mau-2	C (18)	C (15)		C (15)	C (16)	C (16)					C (9)	C (14)	C (12)	C (15)			C (12)	C (15)	IC (12)	C (21)
	Mau-4	C (17)	C (11)	C (12)		C (15)	C (12)	C (19)			C (6)	C (10)	C (10)	IC (17)	C (12)			C (15)	C (14)	IC (13)	C (17)
	Mau-5	C (9)	C (6)	C (8)	C (11)		C (11)					C (7)	C (10)	C (9)	C (12)			C (7)	C (6)	IC (15)	C (10)
	Mau-7	C (15)	C (10)	C (14)	C (11)	C (16)		C (17)			C (13)	C (15)	C (13)	C (11)	C (10)			C (11)	C (12)	IC (12)	C (14)
Mayotte	May-4	C (9)	IC (10)		C (10)		C (12)		C (17)	C (13)	C (12)										
	May-5							C (15)		C (17)	C (11)										
	May-6							C (14)	C (16)												
	May-17	C (14)	C (6)		C (15)		C (15)	C (19)	C (12)												
Tunisia	Tn	C (24)	C (18)	C (24)	C (15)	C (23)	C (22)						C (24)	C (25)	C (17)		C (25)	C (19)	C (19)	C (18)	C (22)
	Sok	C (7)	C (16)	C (22)	C (21)	C (18)	C (17)					C (30)		C (20)	C (18)			C (22)	C (21)	C (23)	C (17)
	Zer11-1	C (12)	C (16)	C (24)	C (18)	C (18)	C (16)					C (17)	C (22)		C (22)			C (18)	C (19)	C (18)	C (20)
	Zer11-2	C (27)	C (18)	C (17)	C (17)	C (21)	C (20)					C (18)	C (14)	C (15)				C (19)	C (17)	C (18)	C (17)
	AinT11-1	C (11)	C (18)														C (12)				
	AinT11-2	C (12)	C (12)									C (15)				C (25)					
Lebanon	Lib-1	C (13)	C (10)	C (12)	C (12)	C (15)	C (16)					C (20)	C (11)	C (16)	C (13)				C (21)	C (12)	
	Lib-2	C (13)	C (14)	C (12)	C (19)	C (17)	C (15)					C (15)	C (15)	C (12)	C (16)			C (14)		C (18)	C (8)
	Lib-3	C (21)	C (12)	C (16)	C (16)	C (14)	C (14)					C (16)	C (10)	C (24)	C (15)			C (19)	C (18)		C (17)
	Lib-4	C (10)	C (16)	C (15)	C (14)	C (10)	C (16)					C (12)	C (19)	C (16)	C (17)				C (11)	C (11)	

Crosses were classified either compatible (C, hatching rate (HR) >90%) or incompatible (IC, HR  = 0%, in bold). The number of egg rafts collected in each cross is bracketed. Boxed crosses were performed between mosquito lines from the same population. *, Crosses corresponding to data from Atyame et al. [17]. Note that crosses between mosquitoes from the same isofemale line are always compatible.

The *w*Pip-III group significantly differed from other groups (*P*  = 0.006), showing higher CI levels (Table 1 and Table S4 in File S1). However, this was estimated from a limited number of crosses between six strains of the *w*Pip-III group and the high CI level mainly pertained to two lines (Sl and Mc), which induce opposed CI patterns when crossed with other *w*Pip groups (Tables S7–S9 in File S1). They both originate from California, where CI was reported in 1980 [27], suggesting that the *w*Pip-III group might be more heterogeneous than measured with polymorphic markers used in this study. On the other hand, the difference between Sl and Mc lines may result from genetic drift since their sampling in 1950 and 1984, respectively [24], [31]. Indeed, *Cx. pipiens* lines can modify their crossing types in only 50 generations in laboratory conditions [29]. Would it be the case, evolution in the laboratory would more likely concern the Sl line since Mc displays CI patterns identical to the *w*Pip-III group line Albu-3 sampled in 2012 from New Mexico (Table S4 and Tables S8–S9 in File S1).

In contrast to intra-group crosses, CI occurred more frequently in crosses between lines infected with different *w*Pip groups (Inter*w*Pip crosses) (mean frequency of compatible crosses of 0.94±0.016 in Intra*w*Pip crosses *vs*. 0.45±0.03 in Inter*w*Pip crosses, *P*<0.001). A significant effect of *w*Pip group combinations on the extent of CI was detected (*P*<0.001). An illustration is the *w*Pip-I group, half-compatible (29/45) with the *w*Pip-II group, fully incompatible (36/36) with the *w*Pip-IV group and fully compatible (10/10) with the *w*Pip-V group (Table 1 and Tables S7 in File S1). Similar results were also obtained in other group combinations. This variability mainly relies on the polymorphism of *w*Pip genomes because the stability of CI properties over *Cx. pipiens* life span observed in previous investigations excluded the role of other factors such as density levels, nuclear background or sperm competitive ability [41], [42]. In addition, males from five *w*Pip infected lines [Tn (*w*Pip-I), Lv (*w*Pip-II), Mc and Sl (*w*Pip-III) and Is (*w*Pip-IV)] displaying incompatibility with infected females always show full compatibility when uninfected [16], [35].

Taken together, these results establish that the genetic proximity of *w*Pip strains correlates with their compatibility. This issue could not be addressed without the knowledge of the *w*Pip phylogeny, only recently worked-out [21]. Although such a correlation was hypothesized, previous surveys in *Drosophila* produced contrasting results: Charlat et al. [43] found compatibility between genetically close *Wolbachia* strains, whilst in other investigations, closely related bacteria appeared totally or partially incompatible [20], [44]. However, comparing our large survey to these previous studies cannot be straightforward since we examined a much higher number of crosses and *w*Pip strains displayed a much lower level of genetic divergence than the strains used in the other studies. Indeed, Charlat et al. [43] compared two *Wolbachia* sister strains considered as genetically identical from analysis of the *wsp* gene only, while in the two other studies, the *Wolbachia* strains were genetically closely related yet showed differences in their *wsp* sequences [20], [44]. By contrast, all *w*Pip strains studied here have strictly identical *wsp* genes and could be only discriminated on the basis of other fast evolving markers such as ankyrin genes and mobile genetic elements including prophages and transposable elements [21].

In conclusion, this analysis shows that except for group III, mosquito lines infected with the same *w*Pip groups have a very high probability to be compatible. By contrast, one cannot predict the CI outcome of crosses between mosquito lines infected with different *w*Pip groups, despite the frequent occurrence of CI.

### Bi-directional CI only Occurs between Mosquito Lines Infected with Divergent *w*Pip Groups

To test if *w*Pip groups also predict CI patterns, incompatible crosses were subdivided in UIC and BIC. BIC was never observed among Intra*w*Pip crosses, all incompatible crosses (6%, n  = 12/203) being UIC. Among the 139 incompatible Inter*w*Pip crosses, 58% (n  = 81) and 42% (n  = 58) were respectively UIC and BIC, and most BIC (n  = 53) involved the *w*Pip-IV group (Figure 1 and Table 1). We found a significant effect of the nature of the *w*Pip group combination on CI patterns (*P*<0.001). For instance, the *w*Pip-I group showed more UIC than BIC with the *w*Pip-II group (n  = 11 vs. n  = 5), only UIC with the *w*Pip-III group (n  = 26) and more BIC than UIC with the *w*Pip-IV group (n  = 34 vs. n  = 2) (Figure 1, Table 1 and Table S7 in File S1). Although generating the highest BIC rates, the *w*Pip-IV group nevertheless showed variable rates, from 27.3% (*w*Pip-II crosses, n  = 22) to 94.4–100% (*w*Pip-I and *w*Pip-III crosses, n  = 36 and n  = 3, respectively). This extends further the heterogeneity in the CI patterns of each *w*Pip group when confronted to other groups.

Our finding that bi-directional CI only affects crosses between genetically different *w*Pip groups corroborates results of previous studies showing bi-directional CI between divergent *Wolbachia* strains [14], [45]. These data fit the model according to which multiple *mod*/*resc* functions control CI patterns in *Wolbachia* infecting *Cx. pipiens* [17]–[19]. Although the *mod*/*resc* functions responsible for mutual compatibility are expected to show little variability within a same *w*Pip group, other *mod/resc* functions not involved in mutual compatibility should be neutral thus more prone to diverge between *w*Pip groups and might occasionally produce BIC.

### *Wolbachia* Genetic Divergence and the Evolution of *mod* and *resc* Properties

*Wolbachia* strains can be characterized by their crossing types (CT) or cytotypes (i.e. compatible, uni-directionally or bi-directionally incompatible) with different strains [39]. Cytotypes can be divided into male CT (*mod* ability) and female CT (*resc* ability). We reported previously that *Wolbachia* strains from the *w*Pip-I group with identical genotypes could nevertheless display distinct male and female CTs when crossed with genetically distant *w*Pip strains [17]. To examine how *mod* and *resc* abilities evolved within and among the five *w*Pip groups, we specifically tested whether cytotypes were distributed at random (i.e. different *w*Pip groups share same cytotypes) or showed preferential distribution into specific *w*Pip groups.

Since we could not reasonably examine by reciprocal crossing the 51 *Cx*. *pipiens* isofemale lines (25 infected with *w*Pip-I strains, 4 with *w*Pip-II, 4 with *w*Pip-III, 16 with *w*Pip-IV and 2 with *w*Pip-V), we used the restricted 4RCT (4-ref-cytotype), corresponding to the combination of four male and four female CTs identified by reciprocal crossing with 4 isofemale lines arbitrarily chosen as references: Lv (*w*Pip-II), Mc and Sl (*w*Pip-III) and Is (*w*Pip-IV). Overall, we identified eight distinct male CTs (i to viii, *mod* abilities, Table 3) and four distinct female CTs (1 to 4, *resc* abilities) combined into fourteen 4RCTs (I to XIV). *w*Pip groups globally displayed fewer *resc* than *mod* abilities, *w*Pip-II being an extreme case with a single *resc* and all different *mod* abilities. Theory predicts that the evolution of *mod* functions should be more constrained by selection than the evolution of *resc* functions [46]. Indeed, changing a *resc* function is counter-selected because it renders the mutant unable to ensure its transmission. On the contrary, changing a *mod* function only makes infected males incompatible with resident strains, which is neutral in a panmictic population because males do not transmit the infection. Consistent with this view, we observed a larger polymorphism of the *mod* than the *resc* function in the data set.

**Table 3:** Summary of 4-ref-cytotypes (4RCTs) and male and female crossing types (*mod* and *resc* abilities) identified among the 51 *Culex pipiens* isofemale lines infected with the five *w*Pip groups.

	4-ref-cytotypes	males crossing types					females crossing types					Distribution of 4RCTs in *w*Pip groups				
		Lv	Mc	Sl	Is	*mod*	Lv	Mc	Sl	Is	*resc*	*w*Pip-I	*w*Pip-II	*w*Pip-III	*w*Pip-IV	*w*Pip-V
	I	C	C	C	C	i	**IC**	**IC**	C	**IC**	1	1				
	II	**IC**	C	**IC**	**IC**	ii	**IC**	**IC**	C	**IC**	1	5				
	III	C	C	C	**IC**	iii	**IC**	**IC**	C	**IC**	1	2				
	IV	**IC**	C	**IC**	**IC**	ii	C	C	C	**IC**	2	2		1		
	V	C	C	C	**IC**	iii	C	C	C	**IC**	2	12	1			2
	VI	C	**IC**	C	**IC**	iv	C	C	C	**IC**	2	1				
	VII	**IC**	**IC**	**IC**	**IC**	v	C	C	C	**IC**	2	1				
	VIII	C	C	**IC**	**IC**	vi	C	C	C	**IC**	2	1	1			
	IX	C	C	**IC**	C	vii	C	C	C	**IC**	2		1	2		
	X	C	C	C	C	i	C	C	C	**IC**	2		1			
	XI	C	C	C	C	i	**IC**	C	**IC**	C	3				7	
	XII	C	C	**IC**	C	vii	**IC**	C	**IC**	C	3				7	
	XIII	**IC**	**IC**	C	C	viii	**IC**	C	**IC**	C	3				2	
	XIV	C	C	C	**IC**	iii	**IC**	**IC**	C	C	4			1		
Total	14					8					4	25	4	4	16	2

The cytotypes were determined based on reciprocal crosses between the 51 isofemale lines and four reference laboratory lines (see text): Lv (*w*Pip-II), Mc and Sl (*w*Pip-III) and Is (*w*Pip-IV). C = compatible cross (all hatching rate, HR >90%); IC = incompatible cross (bolded cells, HR  = 0%).

The 4RCTs were not randomly distributed between the *w*Pip groups (Fisher’s exact test, *P*<0.001): ten 4RCTs were specific to a single group (for example 4RCTs I and XI are specific to *w*Pip-I and *w*Pip-IV, respectively), while four 4RCTs were shared by several groups, such as the 4RCT V shared by *w*Pip-I, *w*Pip-II and *w*Pip-V. The *w*Pip-IV group harbors three specific 4RCTs (Table 3), which makes it significantly different from the others in pairwise comparisons (Fisher’s exact test, *P*-value <0.05 for all four comparisons, only three remaining significant after Hommel’s sequential Bonferroni correction, Table 4).

**Table 4:** *P*-values for the pairwise comparisons of 4-ref-cytotypes (4RCTs) distributions between *w*Pip groups.

	*w*Pip-I	*w*Pip-II	*w*Pip-III	*w*Pip-IV
*w*Pip-II	0.12			
*w*Pip-III	*0.008*	1		
*w*Pip-IV	***8.05*** **×** ***10^−10^***	***0.0014***	***0.0006***	
*w*Pip-V	1	1	0.2	*0.013*

Fisher’s exact tests were computed from the Table 3 data. Significant *P*-values (<0.05) are in italics. *P*-values still significant after Hommel’s sequential Bonferroni correction for multiple testing are bolded.

Analysis of the *mod* and *resc* abilities (Table 3) showed a clear partitioning between *w*Pip groups: *w*Pip-IV displayed exclusively the *resc* 3 ability (16/16) and almost exclusively the *mod* i and *mod* vii abilities (14/16), whereas *w*Pip-I, -II, -III and -V mainly displayed the *resc* 2 ability (26/35) and the *mod* ii and *mod* iii abilities (26/35). The special situation of *w*Pip-IV is consistent with the fact that it is involved in 91.4% of crosses that produced BIC (Table 1).

We further examined the independence between the *mod* and *resc* abilities using their respective frequencies deduced from Table 3. As expected from their linked transmission, the two variables were not independent (Fisher’s exact test, *P*<0.001).

### Worldwide Distribution of *w*Pip Strains does not Correlate with CI Patterns

We recently highlighted a clear spatial structure of *w*Pip groups over *Cx. pipiens* distribution range: *w*Pip-I and *w*Pip-III are largely spread over different continents, whereas *w*Pip-II is restricted to Western Europe, *w*Pip-V to Asia, and *w*Pip-IV sporadically present in Europe, Asia and North Africa [22]. We then asked whether the large geographic distribution of the *w*Pip-I and *w*Pip-III groups could be due to more invasive CI properties. The theory on *Wolbachia* dynamics in a panmictic host population predicts that a strain X can invade a population infected by a strain Y if males X induce CI (*mod^X^* ^+^) and if females X rescue CI induced by most of the males Y (*resc* ^X+,Y+^) [46]. As shown in Table S7 in File S1, when crossed with the *w*Pip-II and *w*Pip-III strains infected females, *w*Pip-I males induced modest CI (28.9% (13/45) and 25.4% (17/67), respectively) while *w*Pip-I females efficiently rescued CI (82.2% (37/45) and 86.6% (58/67), respectively). Almost all of crosses with *w*Pip-IV were bi-directionally incompatible, while crosses with *w*Pip-V were fully compatible, a situation which does not favor invasion in either case. Taken together, this suggests that the large geographic distribution of *w*Pip-I is independent from invasive CI properties. The same conclusion stands for *w*Pip-III (Tables S8–S9 in File S1), which was fully compatible with *w*Pip-V, induced low to moderate CI with *w*Pip-II and *w*Pip-IV females (14.3%, 2/14; 50%, 21/42, respectively) and rescued quite efficiently CI with *w*Pip-II and *w*Pip-IV males (71.4%, 10/14; 61.9%, 26/42, respectively). Therefore, considering that all known *Cx. pipiens* populations are infected by *Wolbachia*, reasons other than CI properties should be invoked to explain the present large distribution of *w*Pip-I and *w*Pip-III. This may be a consequence of passive migration due to human activities, a process shown to be responsible for long-distance gene flow [47]. Alternatively, *w*Pip-I and *w*Pip-III infections might confer selective advantages, e.g. higher female fecundity as is the case with the mosquito *Aedes albopictus* [48], or protection against natural enemies as described in *Drosophila melanogaster* [49], [50].

### Conclusion

In this study, we show a clear correlation between genetic divergence of *Wolbachia* strains infecting *Cx. pipiens* mosquitos and crossing relationships: crosses within same genetic groups were mostly compatible and showed no bi-directional CI. Future investigations using theoretical models like parsimony inference models [18], [19] should help addressing how *mod* and *resc* determinants in each *w*Pip group may interplay to explain the observed phenotypes. This is a critical issue for the development of new control strategies of arthropod disease-vector and pest populations, for which *Wolbachia* are now considered as promising tools [51]. The large database of CI relationships in the *Cx*. *pipiens* complex described here should help identifying candidate genes responsible for CI properties by testing their correlation with distinct *mod* and *resc* abilities groups.

## Supporting Information

File S1 **Supporting file contains Tables S1–S10. Table S1.** ***Culex pipiens*** **isofemale lines.** **Table S2. Reciprocal crosses between isofemale lines infected with** ***w*** **Pip strains from the** ***w*** **Pip-I group.** (**A**) Reciprocal crosses between isofemale lines from La Réunion Island according to Atyame et al [17]. (**B**), Crosses between isofemale lines from Tunisia (Tn), Philippines (Ma-B), France (Bf-A), Grece (Ko), Spain (Ep-A and Ep-B) were performed in previous studies [16], [26] and Cotonou (Cot-A and Cot-B) were performed for this study. Crosses were classified either compatible (C, hatching rate (HR) >90%) or incompatible (IC, HR  = 0%, shaded). The number of egg-rafts collected in each cross is bracketed. Note that crosses between mosquitoes from the same isofemale line are always compatible. **Table S3. Reciprocal crosses between isofemale lines infected with** ***w*** **Pip strains from the** ***w*** **Pip-II group.** Isofemale lines were isolated from samples collected in France (Lv), Brisbane (Au) and Cyprus (Ke-A and Ke-B). All crosses were performed by Duron et al. [16]. C = compatible crosses (HR >90%). The number of egg-rafts collected in each cross is bracketed. **Table S4. Reciprocal crosses between isofemale lines infected with** ***w*** **Pip strains from the** ***w*** **Pip-III group.** Isofemale lines were isolated from samples collected in California (Sl and Mc), New Mexico (Albu-3) and France (Bf-B, Trio-2 and Trio-7). *,Crosses corresponding to data from Duron et al. [16]. Crosses were classified either compatible (C, for HR >90% or incompatible (IC, HR  = 0%, shaded). The number of egg-rafts collected in each cross is bracketed. **Table S5. Reciprocal crosses between isofemale lines infected with** ***w*** **Pip strains from the** ***w*** **Pip-IV group**. (**A** and **B**), reciprocal crosses between the isofemale line Is (from Turkey) and the isofemale lines from Tunisia (Bou-1, Bou-2, Kef-1, Kef-2, Tab-1, Tab-2), from Algeria (Dou-1, Dou-2, Guel-1, Guel-2, Kal-1, Kal-2, Lac-1, Lac-2, Souk-2, Ha) and from Italy (CAA). (**C**), reciprocal crosses between the isofemale lines from Tunisia. C = compatible crosses (HR >90%). The number of egg-rafts collected in each cross is bracketed. **Table S6. Reciprocal crosses between isofemale lines infected with** ***w*** **Pip strains from the** ***w*** **Pip-V group**. These crosses correspond to data from Duron et al. [16] and isofemale lines were from China (Kara-C) and Philippines (Ma-A). C = compatible crosses (HR >90%). The number of egg-rafts collected in each cross is bracketed. **Table S7. Crossing relationships between mosquito lines infected with** ***w*** **Pip strains from the** ***w*** **Pip-I group and lines infected with strains from** ***w*** **Pip-II,** ***w*** **Pip-III,** ***w*** **Pip-IV and** ***w*** **Pip-V groups**. (**A**) Between *w*Pip-I infected males and females infected with *w*Pip-II, *w*Pip-III, *w*Pip-IV and *w*Pip-V groups. (**B**) Between *w*Pip-I infected females and males infected with *w*Pip-II, *w*Pip-III, *w*Pip-IV and *w*Pip-V groups. *, Crosses corresponding to data from Duron et al. [16], [26]. Crosses were classified either compatible (C, HR >90%) or incompatible (IC, HR  = 0%, shaded). Bi-directionally incompatible crosses are underlined. The number of egg-rafts collected in each cross is bracketed. **Table S8. Crossing relationships between mosquito lines infected with** ***w*** **Pip strains from the** ***w*** **Pip-II group and lines infected with strains from** ***w*** **Pip-III,** ***w*** **Pip-IV and** ***w*** **Pip-V groups.** (**A**) Between *w*Pip-II infected males and females infected with *w*Pip-III, *w*Pip-IV and *w*Pip-V groups. (**B**) Between *w*Pip-II infected females and males infected with *w*Pip-III, *w*Pip-IV and *w*Pip-V groups. *, Crosses corresponding to data from Duron et al. [16], [26]. Crosses were classified either compatible (C, HR >90%) or incompatible (IC, HR  = 0%, shaded). Bi-directionally incompatible crosses are underlined. The number of egg-rafts collected in each cross is bracketed. **Table S9. Crossing relationships between mosquito lines infected with** ***w*** **Pip strains from the** ***w*** **Pip-III group and lines infected with strains from** ***w*** **Pip-IV and** ***w*** **Pip-V groups**. (**A**) Between *w*Pip-III infected males and females infected with *w*Pip-IV and *w*Pip-V groups. (**B**) Between *w*Pip-III infected females and males infected with *w*Pip-IV and *w*Pip-V groups. *, Crosses corresponding to data from Duron et al. [16]. Crosses were classified either compatible (C, HR >90%) or incompatible (IC, HR  = 0%, shaded). Bi-directionally incompatible crosses are underlined. The number of egg-rafts collected in each cross is bracketed. **Table S10. Crossing relationships between mosquito lines infected with** ***w*** **Pip strains from the groups** ***w*** **Pip-IV and** ***w*** **Pip-V**. *, Crosses corresponding to data from Duron et al. [16]. IC = incompatible crosses (HR  = 0%, shaded). Bi-directionally incompatible crosses are underlined. The number of egg-rafts collected in each cross is bracketed.
(PDF)
