# Supplementary material for: Wolbachia Divergence and the Evolution of Cytoplasmic Incompatibility in Culex pipiens
Source: PLoS One. 2014 Jan 31;9(1):e87336. doi: 10.1371/journal.pone.0087336 (PMC3909092; doi:10.1371/journal.pone.0087336)
Supplement: File S1 — Supporting file contains Tables S1–S10. Table S1. Culex pipiens isofemale lines. Table S2. Reciprocal crosses between isofemale lines infected with w Pip strains from the w Pip-I group. (A) Reciprocal crosses between isofemale lines from La Réunion Island according to Atyame et al [17]. (B), Crosses between isofemale lines from Tunisia (Tn), Philippines (Ma-B), France (Bf-A), Grece (Ko), Spain (Ep-A and Ep-B) were performed in previous studies [16], [26] and Cotonou (Cot-A and Cot-B) were performed for this study. Crosses were classified either compatible (C, hatching rate (HR) >90%) or incompatible (IC, HR = 0%, shaded). The number of egg-rafts collected in each cross is bracketed. Note that crosses between mosquitoes from the same isofemale line are always compatible. Table S3. Reciprocal crosses between isofemale lines infected with w Pip strains from the w Pip-II group. Isofemale lines were isolated from samples collected in France (Lv), Brisbane (Au) and Cyprus (Ke-A and Ke-B). All crosses were performed by Duron et al. [16]. C = compatible crosses (HR >90%). The number of egg-rafts collected in each cross is bracketed. Table S4. Reciprocal crosses between isofemale lines infected with w Pip strains from the w Pip-III group. Isofemale lines were isolated from samples collected in California (Sl and Mc), New Mexico (Albu-3) and France (Bf-B, Trio-2 and Trio-7). *,Crosses corresponding to data from Duron et al. [16]. Crosses were classified either compatible (C, for HR >90% or incompatible (IC, HR = 0%, shaded). The number of egg-rafts collected in each cross is bracketed. Table S5. Reciprocal crosses between isofemale lines infected with w Pip strains from the w Pip-IV group. (A and B), reciprocal crosses between the isofemale line Is (from Turkey) and the isofemale lines from Tunisia (Bou-1, Bou-2, Kef-1, Kef-2, Tab-1, Tab-2), from Algeria (Dou-1, Dou-2, Guel-1, Guel-2, Kal-1, Kal-2, Lac-1, Lac-2, Souk-2, Ha) and from Italy (CAA). (C), reciprocal crosses betwee [file pone.0087336.s001.pdf]

## Table S1

| Mosquito line | Abbreviation | *w*Pip group | Region | Country | Year of collection | *Culex pipiens* subspecies | Reference |
|---|---|---|---|---|---|---|---|
| Cotonou-A | Cot-A | *w*Pip-I | West Africa | Benin | 2005 | *quinquefasciatus* | Atyame *et al.* (2011a) |
| Cotonou-B | Cot-B | *w*Pip-I | West Africa | Benin | 2005 | *quinquefasciatus* | Atyame *et al.* (2011a) |
| Tunis | Tn | *w*Pip-I | North Africa | Tunisia | 1992 | *pipiens* (f *molestus*) | Duron *et al.* (2005) |
| Sokra | Sok | *w*Pip-I | North Africa | Tunisia | 2007 | *pipiens* | This study |
| Ain Tounga11-1 | AinT11-1 | *w*Pip-I | North Africa | Tunisia | 2009 | *pipiens* | This study |
| Ain Tounga11-2 | AinT11-2 | *w*Pip-I | North Africa | Tunisia | 2009 | *pipiens* | This study |
| Zerga11-1 | Zer11-1 | *w*Pip-I | North Africa | Tunisia | 2009 | *pipiens* | This study |
| Zerga11-2 | Zer11-2 | *w*Pip-I | North Africa | Tunisia | 2009 | *pipiens* | This study |
| Manille-B | Ma-B | *w*Pip-I | Asia | Philippines | 2003 | *quinquefasciatus* | Duron *et al.* (2006) |
| Bifa-A | Bf-A | *w*Pip-I | Europe | France | 2002 | *pipiens* | Duron *et al.* (2006) |
| Kol | Ko | *w*Pip-I | Europe | Grece | 2002 | *pipiens* | Duron *et al.* (2006) |
| El Palmar-A | Ep-A | *w*Pip-I | Europe | Spain | 2005 | *pipiens* | Duron *et al.* (2007) |
| El Palmar-B | Ep-B | *w*Pip-I | Europe | Spain | 2005 | *pipiens* | Duron *et al.* (2007) |
| Liban-1 | Lib-1 | *w*Pip-I | Middle East | Lebanon | 2010 | *pipiens* | Dumas *et al.* (2013) |
| Liban-2 | Lib-2 | *w*Pip-I | Middle East | Lebanon | 2010 | *pipiens* | Dumas *et al.* (2013) |
| Liban-3 | Lib-3 | *w*Pip-I | Middle East | Lebanon | 2010 | *pipiens* | Dumas *et al.* (2013) |
| Liban-4 | Lib-4 | *w*Pip-I | Middle East | Lebanon | 2010 | *pipiens* | Dumas *et al.* (2013) |
| Pie-11 | Pie-11 | *w*Pip-I | Indian Ocean | La Réunion | 2007 | *quinquefasciatus* | Atyame *et al.* (2011b) |
| Su-132 | Su-132 | *w*Pip-I | Indian Ocean | La Réunion | 2007 | *quinquefasciatus* | Atyame *et al.* (2011b) |
| Leu-132 | Leu-132 | *w*Pip-I | Indian Ocean | La Réunion | 2007 | *quinquefasciatus* | Atyame *et al.* (2011b) |
| Leu-118 | Leu-118 | *w*Pip-I | Indian Ocean | La Réunion | 2007 | *quinquefasciatus* | Atyame *et al.* (2011b) |
| Su-118 | Su-118 | *w*Pip-I | Indian Ocean | La Réunion | 2007 | *quinquefasciatus* | Atyame *et al.* (2011b) |
| Leu-58 | Leu-58 | *w*Pip-I | Indian Ocean | La Réunion | 2007 | *quinquefasciatus* | Atyame *et al.* (2011b) |
| Pie-58 | Pie-58 | *w*Pip-I | Indian Ocean | La Réunion | 2007 | *quinquefasciatus* | Atyame *et al.* (2011b) |
| Su-122 | Su-122 | *w*Pip-I | Indian Ocean | La Réunion | 2007 | *quinquefasciatus* | Atyame *et al.* (2011b) |
| Leu-122 | Leu-122 | *w*Pip-I | Indian Ocean | La Réunion | 2007 | *quinquefasciatus* | Atyame *et al.* (2011b) |
| Su-133 | Su-133 | *w*Pip-I | Indian Ocean | La Réunion | 2007 | *quinquefasciatus* | Atyame *et al.* (2011b) |

## Table S1

| Mosquito line | Abbreviation | *w*Pip group | Region | Country | Year of collection | *Culex pipiens* subspecies | Reference |
|---|---|---|---|---|---|---|---|
| Maurice-2 | Mau-2 | *w*Pip-I | Indian Ocean | Mauritius | 2010 | *quinquefasciatus* | Dumas *et al*. (2013) |
| Maurice-4 | Mau-4 | *w*Pip-I | Indian Ocean | Mauritius | 2010 | *quinquefasciatus* | Dumas *et al*. (2013) |
| Maurice-5 | Mau-5 | *w*Pip-I | Indian Ocean | Mauritius | 2010 | *quinquefasciatus* | Dumas *et al*. (2013) |
| Maurice-7 | Mau-7 | *w*Pip-I | Indian Ocean | Mauritius | 2010 | *quinquefasciatus* | Dumas *et al*. (2013) |
| Mayotte-4 | May-4 | *w*Pip-I | Indian Ocean | Mayotte | 2010 | *quinquefasciatus* | Dumas *et al*. (2013) |
| Mayotte-5 | May-5 | *w*Pip-I | Indian Ocean | Mayotte | 2010 | *quinquefasciatus* | Dumas *et al*. (2013) |
| Mayotte-6 | May-6 | *w*Pip-I | Indian Ocean | Mayotte | 2010 | *quinquefasciatus* | Dumas *et al*. (2013) |
| Mayotte-17 | May-17 | *w*Pip-I | Indian Ocean | Mayotte | 2010 | *quinquefasciatus* | Dumas *et al*. (2013) |
| | | | | | | | |
| Australie | Au | *w*Pip-II | Australia | Brisbane | 2004 | Hybrid (*pipiens/quinquefasciatus*) | Duron *et al*. (2005) |
| Keo-A | Ke-A | *w*Pip-II | Europe | Cyprus | 2003 | *pipiens* | Duron *et al*. (2006) |
| Keo-B | Ke-B | *w*Pip-II | Europe | Cyprus | 2003 | *pipiens* | Duron *et al*. (2006) |
| LaVar | Lv | *w*Pip-II | Europe | France | 2003 | *pipiens* | Duron *et al*. (2005) |
| | | | | | | | |
| Slab | Sl | *w*Pip-III | North America | California | 1950 | *quinquefasciatus* | Georghiou *et al*. (1966) |
| MaClo | Mc | *w*Pip-III | North America | California | 1984 | *quinquefasciatus* | Duron *et al*. (2005) |
| Albuquerque | Albu | *w*Pip-III | North America | New Mexico | 2012 | *quinquefasciatus* | This study |
| Triolet-2 | Trio-2 | *w*Pip-III | Europe | France | 2011 | *pipiens* | Dumas *et al*. (2013) |
| Triolet-7 | Trio-7 | *w*Pip-III | Europe | France | 2011 | *pipiens* | Dumas *et al*. (2013) |
| Bifa-B | Bf-B | *w*Pip-III | Europe | France | 2002 | *pipiens* | Duron *et al*. (2006) |
| | | | | | | | |
| Harash | Ha | *w*Pip-IV | North Africa | Algeria | 2006 | *pipiens* | This study |
| Douas-1 | Dou-1 | *w*Pip-IV | North Africa | Algeria | 2008 | *pipiens* | This study |
| Douas-2 | Dou-2 | *w*Pip-IV | North Africa | Algeria | 2008 | *pipiens* | This study |
| Guelma-1 | Guel-1 | *w*Pip-IV | North Africa | Algeria | 2008 | *pipiens* | This study |
| Guelma-2 | Guel-2 | *w*Pip-IV | North Africa | Algeria | 2008 | *pipiens* | This study |
| Kala-1 | Kal-1 | *w*Pip-IV | North Africa | Algeria | 2008 | *pipiens* | This study |
| Kala-2 | Kal-2 | *w*Pip-IV | North Africa | Algeria | 2008 | *pipiens* | This study |

# Table S1

| Mosquito line | Abbreviation | *w*Pip group | Region | Country | Year of collection | *Culex pipiens* subspecies | Reference |
|---|---|---|---|---|---|---|---|
| Lac-1 | Lac-1 | *w*Pip-IV | North Africa | Algeria | 2008 | *pipiens* | This study |
| Lac-2 | Lac-2 | *w*Pip-IV | North Africa | Algeria | 2008 | *pipiens* | This study |
| Souk Ahras-1 | Souk-1 | *w*Pip-IV | North Africa | Algeria | 2008 | *pipiens* | This study |
| Souk Ahras-2 | Souk-2 | *w*Pip-IV | North Africa | Algeria | 2008 | *pipiens* | This study |
| Ain Tounga31-1 | AinT31-1 | *w*Pip-IV | North Africa | Tunisia | 2009 | *pipiens* | This study |
| Ain Tounga31-2 | AinT31-2 | *w*Pip-IV | North Africa | Tunisia | 2009 | *pipiens* | This study |
| Zerga31-1 | Zer31-1 | *w*Pip-IV | North Africa | Tunisia | 2009 | *pipiens* | This study |
| Zerga31-2 | Zer31-2 | *w*Pip-IV | North Africa | Tunisia | 2009 | *pipiens* | This study |
| Boussalem-1 | Bou-1 | *w*Pip-IV | North Africa | Tunisia | 2008 | *pipiens* | This study |
| Boussalem-2 | Bou-2 | *w*Pip-IV | North Africa | Tunisia | 2008 | *pipiens* | This study |
| Kef-1 | Kef-1 | *w*Pip-IV | North Africa | Tunisia | 2008 | *pipiens* | This study |
| Kef-2 | Kef-2 | *w*Pip-IV | North Africa | Tunisia | 2008 | *pipiens* | This study |
| Tabarka-1 | Tab-1 | *w*Pip-IV | North Africa | Tunisia | 2008 | *pipiens* | This study |
| Tabarka-2 | Tab-2 | *w*Pip-IV | North Africa | Tunisia | 2008 | *pipiens* | This study |
| Hang Zhou | Hang | *w*Pip-IV | Asia | China | 2003 | Hybrid (*pipiens/quinquefasciatus*) | This study |
| Istanbul | Is | *w*Pip-IV | Middle East | Turkey | 2003 | *pipiens* (f *molestus*) | Duron *et al*. (2005) |
| CAA | CAA | *w*Pip-IV | Europe | Italy | 1992 | *pipiens* | Calvitti *et al*. (2010) |
| | | | | | | | |
| Kara-C | Ka-C | *w*Pip-V | Asia | China | 2003 | *quinquefasciatus* | Duron *et al*. (2006) |
| Karaoké | Kara | *w*Pip-V | Asia | China | 2003 | *quinquefasciatus* | This study |
| Manille-A | Ma-A | *w*Pip-V | Asia | Philippines | 2003 | *quinquefasciatus* | Duron *et al*. (2006) |

## Table S2

### A

| | males | | | | | | | | | |
|---|---|---|---|---|---|---|---|---|---|---|
| females | Pie-11 | Su-132 | Leu-132 | Leu-118 | Su-118 | Leu-58 | Pie-58 | Su-122 | Leu-122 | Su-133 |
| Pie-11 | | C (6) | C (9) | C (8) | C (8) | C (7) | C (7) | C (8) | C (7) | C (9) |
| Su-132 | C (7) | | C (9) | C (10) | C (6) | C (9) | C (9) | C (12) | C (9) | C (11) |
| Leu-132 | C (7) | C (8) | | C (12) | C (6) | C (8) | C (11) | C (12) | C (8) | C (7) |
| Leu-118 | C (8) | C (7) | C (4) | | C (5) | C (8) | C (7) | C (8) | C (6) | C (5) |
| Su-118 | C (7) | C (7) | C (7) | C (5) | | C (6) | C (9) | C (6) | C (3) | C (4) |
| Leu-58 | C (9) | C (11) | C (5) | C (9) | C (10) | | C (4) | C (7) | C (5) | C (10) |
| Pie-58 | C (9) | C (10) | C (7) | C (10) | C (8) | C (12) | | C (7) | C (13) | C (7) |
| Su-122 | C (6) | C (8) | C (5) | C (6) | C (6) | C (7) | C (8) | | C (7) | C (8) |
| Leu-122 | C (10) | C (7) | C (8) | C (5) | C (13) | C (6) | C (12) | C (8) | | C (9) |
| Su-133 | C (6) | C (4) | C (4) | C (7) | C (4) | C (8) | C (5) | C (5) | C (5) | |

### B

| | males | | | | | | | |
|---|---|---|---|---|---|---|---|---|
| females | Cot-A | Cot-B | Tn | Ma-B | Bf-A | Ko | Ep-A | Ep-B |
| Cot-A | | C (18) | C (8) | | | | | |
| Cot-B | C (17) | | C (2) | | | C (13) | | |
| Tn | C (21) | C (9) | | C (14) | C (27) | C (33) | | C (23) |
| Ma-B | | | C (6) | | C (11) | C (8) | | |
| Bf-A | | | C (16) | C (11) | | C (21) | C (21) | C (14) |
| Ko | | C (10) | C (15) | C (8) | C (11) | | C (12) | |
| Ep-A | | | | | C (16) | C (8) | | C (3) |
| Ep-B | | | C (30) | | IC (26) | | C (15) | |

## Table S3

| females | males | | | |
|---|---|---|---|---|
| | Lv | Au | Ke-A | Ke-B |
| Lv | | C (20) | C (19) | C (13) |
| Au | C (23) | | C (11) | C (18) |
| Ke-A | C (15) | C (3) | | C (15) |
| Ke-B | C (14) | C (21) | C (8) | |

# Table S4

| *w*Pip-III females | *w*Pip-III males | | | | | |
|---|---|---|---|---|---|---|
| | Sl | Mc | Bf-B | Trio-2 | Trio-7 | Albu-3 |
| Sl | | IC (99)* | IC (27)* | C (23) | C (22) | IC (19) |
| Mc | C (43)* | | C (11)* | | C (23) | C (12) |
| Bf-B | C (15)* | C (9)* | | | | |
| Trio-2 | C (11) | | | | C (7) | |
| Trio-7 | C (9) | IC (14) | | C (13) | | |
| Albu-3 | C (22) | C (13) | | | | |

## Table S5

### A

| | Females |
|---|---|
| Males | Is |
| Bou-1 | C (19) |
| Bou-2 | C (19) |
| Kef-1 | C (13) |
| Kef-2 | C (18) |
| Tab-1 | C (11) |
| Tab-2 | C (21) |
| Dou-1 | C (16) |
| Dou-2 | C (16) |
| Guel-1 | C (21) |
| Guel-2 | C (12) |
| Kal-1 | C (12) |
| Kal-2 | C (10) |
| Lac-1 | C (12) |
| Lac-2 | C (21) |
| Souk-2 | C (14) |
| Ha | C (17) |
| C AA | C (12) |

### B

| | Males |
|---|---|
| Females | Is |
| Bou-1 | C (15) |
| Bou-2 | C (11) |
| Kef-1 | C (26) |
| Kef-2 | C (22) |
| Tab-1 | C (12) |
| Tab-2 | C (13) |
| Dou-1 | C (23) |
| Dou-2 | C (14) |
| Guel-1 | C (20) |
| Guel-2 | C (24) |
| Kal-1 | C (15) |
| Kal-2 | C (20) |
| Lac-1 | C (6) |
| Lac-2 | C (18) |
| Souk-2 | C (13) |
| Ha | C (15) |
| C AA | C (18) |

### C

| | males | | | |
|---|---|---|---|---|
| females | Zer31-1 | Zer31-2 | AinT31-1 | AinT31-2 |
| Zer31-1 | | C (8) | | |
| Zer31-2 | C (12) | | | |
| AinT31-1 | | | | C (18) |
| AinT31-2 | | | C (17) | |

## Table S6

| | males | |
|---|---|---|
| females | Ka-C | Ma-A |
| Ka-C | | C (8) |
| Ma-A | C (6) | |

# Table S7

## A

| | females | | | | | | | | | | | |
|---|---|---|---|---|---|---|---|---|---|---|---|---|
| | *w* Pip-II | | | | *w* Pip-III | | | *w* Pip-IV | | | *w* Pip-V | |
| *w* Pip-I males | Lv | Au | Ke-A | Ke-B | Sl | Mc | Bf-B | Is | Ha | CAA | Ka-C | Ma-A |
| Bf-A | C (10)* | C (5)* | C (15)* | C (9)* | C (18)* | IC (14)* | IC (31)* | IC (5)* | | | C (10)* | C (13)* |
| Ko | C (17)* | C (11)* | C (17)* | C (9)* | C (39)* | C (9)* | | IC (26)* | | | C (9)* | C (6)* |
| Ep-A | IC (11)* | IC (12)* | C (8)* | C (7)* | C (9)* | | C (28)* | IC (18)* | | | C (15)* | C (10)* |
| Ep-B | C (17)* | IC (16)* | | C (18)* | C (33)* | C (15)* | C (29)* | IC (21)* | | | C (14)* | C (11)* |
| Ma-B | IC (7)* | IC (11)* | C (10)* | | IC (19)* | IC (14)* | IC (18)* | IC (36)* | | | C (14)* | C (12)* |
| Lib-1 | C (12) | | | | C (18) | C (22) | | IC (14) | | | | |
| Lib-2 | | | | | | C (33) | | IC (30) | | | | |
| Lib-3 | C (11) | | | | | IC (19) | | | | | | |
| Lib-4 | C (17) | | | | C (21) | | | IC (19) | | | | |
| Tn | IC (21)* | IC (10)* | C (6)* | C (17)* | IC (31)* | C (19)* | C (17)* | IC (30)* | IC (8) | | | |
| Sok | C (16) | | | | C (19) | C (18) | | IC (18) | | | | |
| AinT11-1 | C (26) | | | | C (12) | C (12) | | IC (12) | | | | |
| AinT11-2 | C (18) | | | | C (18) | C (12) | | IC (13) | | | | |
| Zer11-1 | C (15) | | | | IC (32) | C (24) | | IC (19) | | | | |
| Zer11-2 | C (24) | | | | C (18) | C (16) | | IC (12) | | | | |
| Cot-A | | | | | IC (14) | | | IC (15) | | | | |
| Cot-B | | | | | IC (19) | | | IC (14) | | | | |
| Pie-11 | C (11)* | | | | C (18)* | C (15)* | | C (45)* | | IC (22) | | |
| Su-132 | IC (12)* | | | | IC (22)* | C (13)* | | IC (19)* | | | | |
| Leu-132 | IC (12)* | | | | IC (22)* | C (10)* | | IC (16)* | | IC (24) | | |
| Leu-118 | C (7)* | | | | C (17)* | C (10)* | | IC (12)* | | | | |
| Su-118 | IC (12)* | | | | IC (28)* | C (11)* | | IC (16)* | | | | |
| Leu-58 | C (9)* | | | | C (12)* | C (14)* | | IC (8)* | | | | |
| Pie-58 | IC (12)* | | | | IC (21)* | C (15)* | | IC (12)* | | | | |
| Su-122 | C (12)* | | | | C (12)* | C (21)* | | IC (14)* | | | | |
| Leu-122 | IC (18)* | | | | IC (29)* | C (15)* | | IC (15)* | | | | |
| Su-133 | IC (9)* | | | | IC (24)* | C (13)* | | IC (17)* | | | | |
| Mau-2 | | | | | IC (20) | C (23) | | | | | | |
| Mau-4 | | | | | C (23) | C (19) | | IC (21) | | | | |
| Mau-5 | C (14) | | | | C (24) | C (17) | | IC (18) | | | | |
| Mau-7 | C (15) | | | | C (23) | C (14) | | IC (24) | | | | |
| May-4 | C (19) | | | | C (21) | | | IC (13) | | | | |
| May-5 | | | | | | C (23) | | IC (14) | | | | |
| May-6 | C (17) | | | | C (34) | C (22) | | IC (23) | | | | |
| May-17 | C (22) | | | | C (21) | C (20) | | IC (17) | | | | |

## B

| | males | | | | | | | | | | | |
|---|---|---|---|---|---|---|---|---|---|---|---|---|
| | *w* Pip-II | | | | *w* Pip-III | | | *w* Pip-IV | | | *w* Pip-V | |
| *w* Pip-I females | Lv | Au | Ke-A | Ke-B | Sl | Mc | Bf-B | Is | Ha | CAA | Ka-C | Ma-A |
| Bf-A | C (21)* | C (35)* | C (5)* | C (26)* | C (12)* | C (14)* | C (18)* | IC (15)* | | | C (20)* | C (28)* |
| Ko | C (19)* | C (12)* | C (13)* | C (13)* | C (30)* | C (24)* | | IC (50)* | | | C (7)* | C (22)* |
| Ep-A | C ( 11)* | C (9)* | C (10)* | C (24) | C (9)* | | C (9)* | IC (27)* | | | C (19)* | C (9)* |
| Ep-B | C (7)* | C (4)* | | C (6)* | C (7)* | C (10)* | C (3)* | IC (22)* | | | C (12)* | C (11)* |
| Ma-B | C (8)* | C (11)* | C (6)* | | C (11)* | C (24)* | C (8)* | IC (8)* | | | C (11)* | C (6)* |
| Lib-1 | C (20) | | | | C (9) | C (21) | | IC (6) | | | | |
| Lib-2 | | | | | | C (21) | | IC (16) | | | | |
| Lib-3 | C (16) | | | | | C (12) | | | | | | |
| Lib-4 | C (11) | | | | C (10) | | | IC (8) | | | | |
| Tn | C (35)* | C (13)* | C (15)* | C (17)* | C (66)* | C (54)* | C (20)* | IC (35)* | C (9) | | | |
| Sok | C (20) | | | | C (20) | C (15) | | IC (15) | | | | |
| AinT11-1 | C (11) | | | | C (11) | C (10) | | IC (12) | | | | |
| AinT11-2 | C (12) | | | | C (12) | C (18) | | IC (10) | | | | |
| Zer11-1 | C (16) | | | | C (16) | C (16) | | IC (12) | | | | |
| Zer11-2 | C (12) | | | | C (12) | C (15) | | IC (10) | | | | |
| Cot-A | | | | | C (13) | | | IC (6) | | | | |
| Cot-B | | | | | C (16) | | | IC (6) | | | | |
| Pie-11 | IC (4)* | | | | C (13)* | IC (5)* | | IC (13)* | | IC (42) | | |
| Su-132 | IC (9)* | | | | C (7)* | IC (10)* | | IC (11)* | | | | |
| Leu-132 | IC (12)* | | | | C (12)* | IC (7)* | | IC (10)* | | | | |
| Leu-118 | IC (9)* | | | | C (5)* | IC (12)* | | IC (7)* | | IC (40) | | |
| Su-118 | C (3)* | | | | C (7)* | C (9)* | | IC (9)* | | | | |
| Leu-58 | C (32)* | | | | C (10)* | C (30)* | | IC (12)* | | | | |
| Pie-58 | IC (6)* | | | | C (9)* | IC (7)* | | IC (9)* | | | | |
| Su-122 | IC (8)* | | | | C (6)* | IC (9)* | | IC (5)* | | | | |
| Leu-122 | IC (6)* | | | | C (5)* | IC (6)* | | IC (6)* | | | | |
| Su-133 | IC (6)* | | | | C (7)* | IC (5)* | | IC (6)* | | | | |
| Mau-2 | | | | | C (13) | C (12) | | | | | | |
| Mau-4 | | | | | C (8) | IC (10) | | IC (13) | | | | |
| Mau-5 | C (13) | | | | C (7) | C (4) | | IC (11) | | | | |
| Mau-7 | C (14) | | | | C (10) | C (13) | | IC (16) | | | | |
| May-4 | C (6) | | | | C (14) | | | IC (11) | | | | |
| May-5 | | | | | | C (16) | | IC (16) | | | | |
| May-6 | C (15) | | | | C (16) | C (11) | | IC (23) | | | | |
| May-17 | C (18) | | | | C (16) | C (14) | | IC (18) | | | | |

## Table S8

### A

| females | | *w*Pip-II males: Lv | Au | Ke-A | Ke-B |
|---|---|---|---|---|---|
| *w*Pip-III | Sl | IC (30)* | IC (34)* | C (14)* | C (19)* |
| | Mc | C (36)* | C (9)* | C (17)* | C (9)* |
| | Bf-B | C (18)* | C (12)* | C (17)* | IC (28)* |
| | Trio-2 | IC (10) | | | |
| | Albu-3 | C (13) | | | |
| *w*Pip-IV | Is | IC (40)* | C (11)* | IC (12)* | C (5)* |
| | Bou-1 | IC (14) | | | |
| | Bou-2 | IC (6) | | | |
| | Kef-1 | IC (19) | | | |
| | Kef-2 | IC (12) | | | |
| | Tab-1 | IC (12) | | | |
| | Tab-2 | IC (19) | | | |
| | Dou-1 | IC (14) | | | |
| | Dou-2 | IC (24) | | | |
| | Guel-1 | IC (12) | | | |
| | Guel-2 | IC (13) | | | |
| | Kal-1 | IC (18) | | | |
| | Lac-1 | IC (17) | | | |
| | Lac-2 | IC (14) | | | |
| | Souk-1 | IC (12) | | | |
| | Souk-2 | IC (14) | | | |
| | Ha | IC (15) | | | |
| | Hang | IC (18) | | | |
| | CAA | IC (45) | | | |
| *w*Pip-V | Ka-C | C (9)* | C (13)* | C (6)* | IC (23)* |
| | Ma-B | C (8)* | C (11)* | C (6)* | |

### B

| males | | *w*Pip-II females: Lv | Au | Ke-A | Ke-B |
|---|---|---|---|---|---|
| *w*Pip-III | Sl | C (8)* | C (12)* | C (5)* | C (12)* |
| | Mc | C (10)* | C (9)* | C (9)* | C (23)* |
| | Bf-B | IC (17)* | IC (14)* | C (11)* | C (11)* |
| | Trio-2 | C (17) | | | |
| | Albu-3 | C (19) | | | |
| *w*Pip-IV | Is | IC (26)* | IC (7)* | IC (15)* | IC (10)* |
| | Bou-1 | C (17) | | | |
| | Bou-2 | C (12) | | | |
| | Kef-1 | C (16) | | | |
| | Kef-2 | C (20) | | | |
| | Tab-1 | C (14) | | | |
| | Tab-2 | C (24) | | | |
| | Dou-1 | C (13) | | | |
| | Dou-2 | C (18) | | | |
| | Guel-1 | C (21) | | | |
| | Guel-2 | C (21) | | | |
| | Kal-1 | C (13) | | | |
| | Lac-1 | C (18) | | | |
| | Lac-2 | C (15) | | | |
| | Souk-1 | IC (14) | | | |
| | Souk-2 | IC (16) | | | |
| | Ha | C (5) | | | |
| | Hang | IC (23) | | | |
| | CAA | IC (17) | | | |
| *w*Pip-V | Ka-C | C (4)* | C (7)* | C (23)* | C (7)* |
| | Ma-B | IC (7)* | IC (11)* | C (10)* | |

## Table S9

### A

| females | | *w*Pip-III males | | | | |
|---|---|---|---|---|---|---|
| | | Sl | Mc | Bf-B | Trio-7 | Albu-3 |
| *w*Pip-IV | Is | IC (34)* | C (31)* | IC (11)* | IC (18)* | C (11) |
| | Bou-1 | IC (16) | C (12) | | | |
| | Bou-2 | IC (22) | C (13) | | | |
| | Kef-1 | IC (22) | C (14) | | | |
| | Kef-2 | IC (14) | C (16) | | | |
| | Tab-1 | IC (15) | C (16) | | | |
| | Tab-2 | IC (12) | C (12) | | | |
| | Dou-1 | IC (20) | C (22) | | | |
| | Dou-2 | IC (18) | C (18) | | | |
| | Guel-1 | IC (12) | C (13) | | | |
| | Guel-2 | IC (10) | C (18) | | | |
| | Kal-1 | IC (12) | C (10) | | | |
| | Kal-2 | IC (24) | C (9) | | | |
| | Lac-1 | IC (16) | C (15) | | | |
| | Lac-2 | IC (11) | C (12) | | | |
| | Souk-1 | IC (13) | C (12) | | | |
| | Souk-2 | IC (18) | C (12) | | | |
| | Ha | IC (15) | C (11) | | | |
| | Hang | | C (24) | | | |
| | CAA | IC (33) | C (22) | | | |
| *w*Pip-V | Ka-C | C (14)* | C (20)* | C (11)* | | |
| | Ma-A | C (4)* | C (8)* | C (6)* | | |

### B

| males | | *w*Pip-III females | | | | |
|---|---|---|---|---|---|---|
| | | Sl | Mc | Bf-B | Trio-7 | Albu-3 |
| *w*Pip-IV | Is | C (33)* | IC (53)* | IC (27)* | IC (9)* | IC (14) |
| | Bou-1 | IC (25) | C (18) | | | |
| | Bou-2 | IC (19) | C (16) | | | |
| | Kef-1 | IC (13) | C (17) | | | |
| | Kef-2 | C (12) | C (14) | | | |
| | Tab-1 | C (21) | C (14) | | | |
| | Tab-2 | C (14) | C (18) | | | |
| | Dou-1 | C (13) | C (20) | | | |
| | Dou-2 | C (24) | C (24) | | | |
| | Guel-1 | IC (20) | C (23) | | | |
| | Guel-2 | IC (24) | C (22) | | | |
| | Kal-1 | C (12) | C (17) | | | |
| | Kal-2 | IC (17) | C (16) | | | |
| | Lac-1 | C (17) | C (18) | | | |
| | Lac-2 | IC (14) | C (18) | | | |
| | Souk-1 | C (16) | IC (17) | | | |
| | Souk-2 | C (23) | IC (16) | | | |
| | Ha | IC (12) | C (10) | | | |
| | Hang | | IC (18) | | | |
| | CAA | C (30) | IC (26) | | | |
| *w*Pip-V | Ka-C | C (23)* | C (4)* | C (25)* | | |
| | Ma-A | C (26)* | C (16)* | C (25)* | | |

## Table S10

| females | | males | | | | |
|---|---|---|---|---|---|---|
| | | *w*Pip-IV | | *w*Pip-V | | |
| | | Is | Hang | Ka-C | Ma-B | Kara |
| *w*Pip-IV | Is | | | IC (7)* | IC (6)* | |
| | Hang | | | | | IC (13) |
| *w*Pip-V | Ka-C | IC (7)* | | | | |
| | Ma-B | IC (7)* | | | | |
| | Kara | | IC (13) | | | |
